# Spectrophotometric Determination of Alfuzosin HCl in Pharmaceutical Formulations with some Sulphonephthalein Dyes

**Published:** 2006-09

**Authors:** Safwan Ashour, M. Fawaz Chehna, Roula Bayram

**Affiliations:** 1 *Department of Chemistry, Faculty of Sciences, Aleppo University, Aleppo, Syria;*; 2 *Department of Pharmaceutical Chemistry and Drug Control, Faculty of Pharmacy, Aleppo University, Aleppo, Syria*

**Keywords:** spectrophotometry, alfuzosin hydrochloride, bromophenol blue, bromocresol purple, bromothymol blue, ion-association complex

## Abstract

Bromocresol purple (BCP), bromophenol blue (BPB) and bromothymol blue (BTB) were used to determine alfuzosin hydrochloride either in pure form or in pharmaceutical formulations. Alfuzosin was extracted as an ion-pair complex from sample solution containing KCl-HCl buffer pH2.2, 2.4 and 2.6 into CHC_l3_ and the absorbance was measured at 407, 413 and 412nm with use of the cited reagents, respectively. The analytical parameters and their effects on the reported systems are investigated. The reactions were extremely rapid at room temperature and the absorbance values remains unchanged up to 24 h. Beer’s law was obeyed in the concentration ranges 1.20-38.3, 0.85-46.0 and 0.63-34.0 μg/ml and detection limits were 0.28, 0.24 and 0.18 μg/ml with BCP, BPB and BTB, respectively. Recoveries were 98.80-101.33%. Interferences of the other ingredients and excipients were not observed. The proposed method is simple, fast and sensitive, and the first reported extractive method for the determination of alfuzosin in commercial tablets.

## INTRODUCTION

Alfuzosin hydrochloride is an alpha_1_-adrenoreceptor blocker. It is used in the symptomatic treatment of urinary obstruction caused by benign prostatic hyperplasia and has been tried in the treatment of hypertension. Alfuzosin hydrochloride is chemically designated as N-{3-[(4-Amino-6,7-dimethoxyquinazolin-2-yl(methyl)amino]propyl}tetrahydro-2-furamide hydrochloride (1).

Few analytical methods have been reported for the determination of alfuzosin in pharmaceuticals and biological fluids. Alfuzosin was determined in human plasma by HPLC-tandem mass spectrometry (2), chiral mobile phase HPLC for chiral separation of three new enantiomers of alfuzosin, doxazosin and terazosin (3) and HPLC methods using fluorescence detection (4-8). Voltammetric methods (cyclic, linear sweep, differential pulse and square-wave voltammetry) were developed for the determination of the alfuzosin in tablet dosage form, human serum and simulated gastric juice at a glassy carbon disk electrode in Britton-Robinson and phosphate buffers over the pH range 2-7.5. Detection limits were found to be 1.56 × 10^-7^M for differential pulse voltammetry and 6.2 × 10^-8^M for square-wave voltammetry (9).

Extractive spectrophotometric procedures are popular for their sensitivity in the assay of drugs and, therefore, ion-pair extractive spectrophotometry has received considerable attention for the quantitative determination of many pharmaceutical compounds (10-13).

However, no reports have appeared dealing with the extractive spectrophotometric method for the determination of alfuzosin in drug forms so far. Therefore, this paper proposes three simple and sensitive extractive spectrophotometric methods for the assay of alfuzosin hydrochloride. The methods are based on ion-pair complexes of drug with dyestuffs such as bromocresol purple (BCP), bromothymol blue (BTB) and bromophenol blue (BPB) and subsequent extraction into chloroform under reaction conditions used.

## EXPERIMENTAL

### Apparatus

A Jasco V-530 UV–VIS spectrophotometer (Japan) with 1 cm quartz cells was used for all absorbance measurements under the following operating conditions: scan speed medium (400nm/min), scan range 350-500 nm and slit width 2nm. Spectra were automatically obtained by Jasco system software. pH measurements were made with Suntex-SP 701 (Taiwan) with combined glass pH electrode.

### Materials

**Pure sample:** Alfuzosin hydrochloride (AFZ), working standard, (C_19_H_27_N_5_O_4_.HCl=425.9 g/mole, obtained from FARMAK, Czech Rebuplic). Its purity was found to be 99.8% according to the compendial method (Titrimetric manufacturer procedure supplied by FARMAK, Pers. Commun.).

**Market samples:** Alfosin 2.5 and 5 mg Tablet (B.P.I.), each tablet was labeled to contain alfuzosin hydrochloride 2.5 mg and 5 mg, respectively, and Alfuzosine Tablet (Al-Saad), each tablet was labeled to contain alfuzosin hydrochloride 5mg.

### Reagents and chemicals

All chemicals were of analytical reagent grade of Merck unless otherwise specified.

1. Hydrochloric acid and potassium chloride, 0.2 M solutions in doubly distilled water.
2. Buffer solutions of required pH were prepared by mixing appropriate volumes of 0.2 M KCl and 0.2 M HCl.
3. BCP, BPB (BDH) and BTB 10^-3^ M solutions in water, freshly prepared.

### Standard stock solution

Standard stock solution must be freshly prepared for cited drug. 10^-1^ M (425.9 μg/ml) of AFZ was prepared in water. The working standard solutions were then prepared by suitable dilution of the stock solution with water.

### Procedure for the assay of bulk sample

Into a series of 50 ml separating funnels, 2 ml of buffer solution of pH2.2 (pH2.4 or 2.5 ml of pH2.6) and 3 ml of BCP (5 ml of BPB or 6 ml of BTB) were placed. An appropriate volume of 10^-3^ M standard drug solution (0.028-0.90 ml for BCP; 0.020-1.08 ml for BPB; 0.015-0.80 ml for BTB) was added to each funnel and mixed well. The funnels were shaken vigorously with 10ml chloroform for 2 min, then allowed to stand for clear separation of the two phases. The separated organic phase was transferred to a 50 ml beaker, dried over anhydrous sodium sulfate, and transferred to a 10 ml volumetric flask. Then the combined extract was made up to the mark with the solvent and mixed. The absorbance of the organic phase was measured at 407, 413 and 412 nm for BCP, BPB and BTB, respectively, against a reagent blank similarly prepared. The standard calibration plot was prepared to calculate the amount of the analyst drug in unknown samples. The colour is stable for at least 24 hrs up to 30°C.

### Procedure for formulations

Twenty tablets containing AFZ.HCl were weighed and finely powdered. An accurately weighed portion of the powder equivalent to 25 mg of AFZ was dissolved in a 25 ml of methanol and mixed for about 5 minutes and then filtered. The methanol was evaporated to about the dryness. The remaining portion of solution was diluted in a 25 ml volumetric flask to the volume with double distilled water. The general procedure was then followed in the concentration ranges mentioned above.

## RESULTS AND DISCUSSION

**Figure 1:**
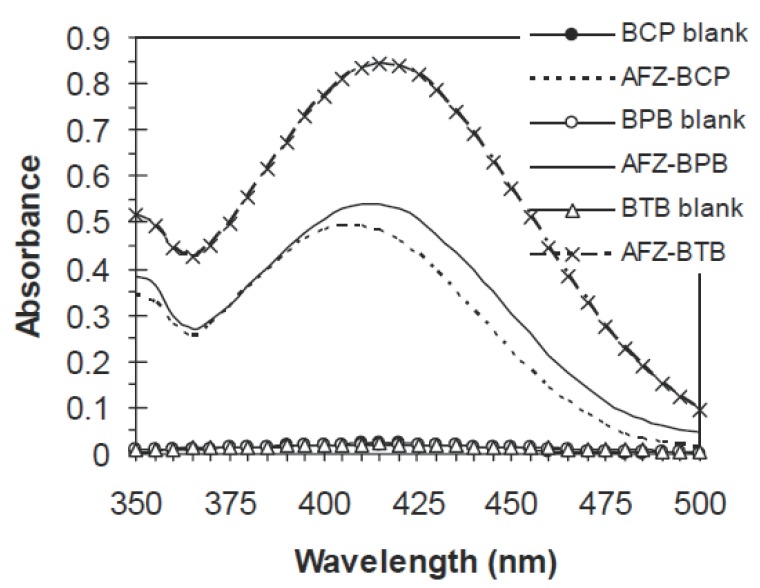
Absorption spectra of AFZ-dye complexes extracted into 10ml chloroform: AFZ=9 μg/ml+BCP, AFZ=11 μg/ml+BPB and AFZ=14 μg/ml+BTB, against their respective blanks.

Alfuzosin hydrochloride forms ion-pair complexes in acidic buffer with dyestuffs such as BCP, BPB and BTB, and these complexes are quantitatively extracted into chloroform. The absorption spectra of the ion-pair complexes extracted into chloroform are shown in Fig. 1. The ion-pair complexes with BCP, BPB and BTB absorbed maximally at 407, 413 and 412 nm, respectively. The colorless reagent blanks under similar conditions showed no absorption.

**Figure 2:**
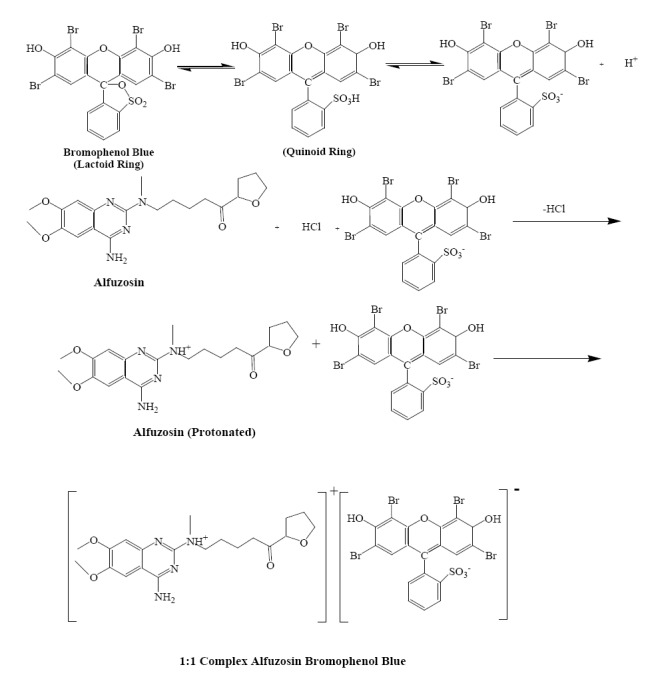
The possible reaction mechanisms.

Alfuzosin hydrochloride contains tertiary amino group which is protonated in acid medium, while sulphonic acid group is present in BTB: that is the only group undergoing dissociation in the pH range 1-5. BPB and BCP are examples of sulphonephthalein type of dye. The colour of such dyes is due to the opening of lactoid ring and subsequent formation of quinoid group. It is supposed that the two tautomers are present in equilibrium but due to strong acidic nature of the sulphonic acid group, the quinoid body must predominate. Finally, the protonated alfuzosin hydrochloride forms ion-pairs with the dyestuffs which are quantitatively extracted into chloroform. The possible reaction mechanisms are proposed and given in Fig 2.

### Optimization of variables

Optimum conditions necessary for rapid and quantitative formation of colored ion-pair complexes with maximum stability and sensitivity were established by a number of preliminary experiments. KCl–HCl buffer (Clark & Lubs buffer) was found to be suitable for all methods. Chloroform was preferred to other solvents (Carbon tetrachloride, dichloromethane, and ether) for its selective and quantitative extraction. Optimum conditions were fixed by varying one parameter at a time while keeping other parameters constant and observing its effect on the absorbance at 407, 413 and 412 nm for BCP, BPB and BTB, respectively.

For BCP, effect of pH was studied by extracting the colored complex species at different pH. Maximal absorbances were observed at the pH2.2 and 3.2 using 0.5-3 ml of buffer. Therefore, 2 ml portions of buffer of pH2.2 were used in all measurements. Similarly, for BPB, a constant absorbance was observed over the pH range 2.2-2.6 using 0.5-3 ml of buffer. Therefore, 2 ml portions of buffer of pH2.4 were used. For BTB, Maximal absorbance was observed at the pH2.6 using 0.5-3 ml of buffer. Therefore, 2.5 ml portions of buffer of pH2.6 were used.

A volume of 3 ml, 5 ml or 6 ml of 10^-3^ M BCP, BPB or BTB was found to be optimal for complete complexation, since the absorbance was found to be maxima at the mentioned volumes.

### Stoichiometric relationship

**Figure 3:**
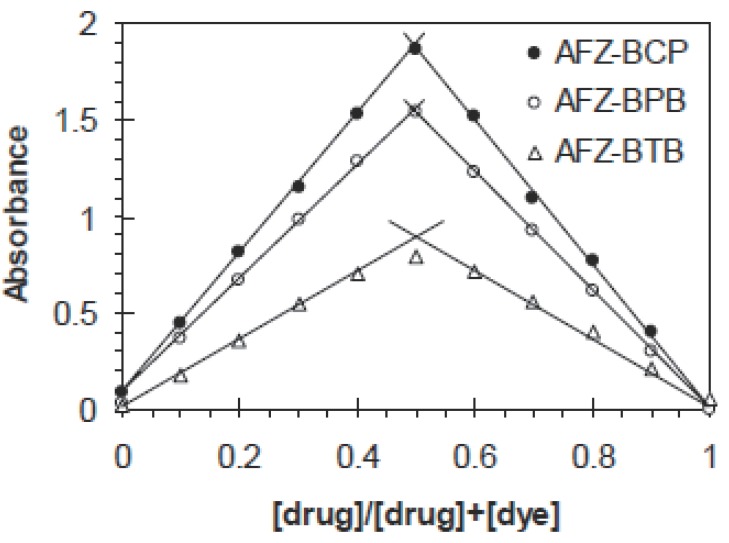
Job’s method of continuous variation of AFZ-dye systems. [AFZ+dye]= 2.10^-4^ M.

**Figure 4:**
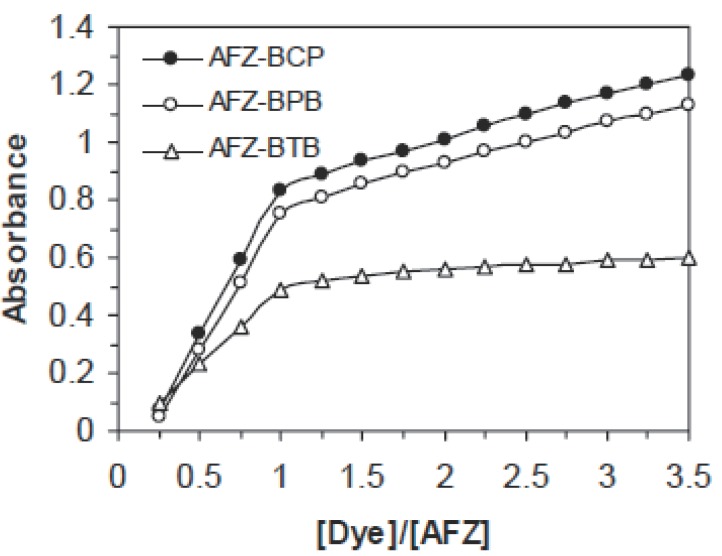
Mole-ratio method of AFZ-dye ion-pair complexes (BPB, BCP and BTB 10^-3^M and AFZ=5 × 10^-5^ M).

The stoichiometric ratio of the drug to dye in each of the colored complexes was determined using the molar ratio (14) and continuous variation (15) methods. It is apparent from the data that ion pair complexes with drug to dye ratio 1:1 are formed (Fig. 3 and 4). The logarithmic formation constants of the formed complexes are calculated from the Harvey and Manning method (16) using the data of the molar ratio and continuous variation methods (Table 1).

**Table 1:** Optical and regression characteristics, precision and accuracy of the proposed methods

Parameters	Extraction method with		
	BCP	BPB	BTB

λmax (nm)	407	413	412
Logarithmic formation constants	6.61	6.53	6.50
Beer’s law limit (μg.ml^-1^)	1.2-38.3	0.85-46.0	0.63-34.0
Molar absorptivity (l.mol^-1^.cm^-1^)	2.41 × 10^4^	2.20 × 10^4^	2.60 × 10^4^
Sandell’s sensitivity (μg.cm^-2^ per 0.001 absorbance unit)	0.034	0.038	0.032
Linear Regression equation^a^	*m=0.0478, b=0.0763*	*m=0.0459, b=0.0299*	*m=0.0612, b=0.0321*
Correlation coefficient (r)	0.9998	0.9999	0.9999
Ringbom optimum concentration range (μg.ml^-1^)	4.0-24.0	2.85-34.0	1.6-24.0
Detection limits (μg.ml^-1^)	0.28	0.24	0.18
Recovery %	99.84 ± 0.66	99.98 ± 0.82	100.94 ± 0.42

a With respect to *A=mC+b*, where C is the concentration (μg ml^-1^) and *A* is absorbance.

### Linearity and range

The Beer’s law limits, molar absorptivity, Sandell’s sensitivity, linear regression equation, correlation coefficient, Ringbom optimum concentration range (*T%=f (logC)*, where *C* in μg/ml) (17) and detection limit determined for each method are given in Table 1. A linear relationship was found between the absorbance at λ_max_ and the concentration of the drug in the ranges 1.2-38.3, 0.85-46.0 and 0.63-34.0 μg.ml^-1^ for BCP, BPB and BTB method, respectively, in the final measured volume of 10ml. Regression analysis of the Beer’s law plots at λ_max_ reveals a good correlation. The graphs show negligible intercept and are described by the regression equation, *A= mC + b* (where *A* is the absorbance of 1 cm layer, *m* is the slope, *b* is the intercept and *C* is the concentration of the measured solution in μg ml^-1^) obtained by the least-squares method (18). The high molar absorptivities of the resulting colored complexes indicate the high sensitivity of the methods.

The detection limit for the proposed method was calculated by using the following relationship (19):

Detection Limite=So2n−2tn−1b

where *n*=number of samples; *b*=slope of line of regression; *t*=student’s *t*-value at 95% confidence level (20);

$$S_{o}^{2}=\mathrm{Variance}=\sum {\left({A-A}_{\mathrm{calc}.}\right)}^{2}/n-2$$

(*A*=experimental value of absorbance; *A_calc_*=absorbance value calculated from the regression equation).

### Validation of the methods

The validity of the proposed methods for the analysis of AFZ has been tested. The results obtained are summarized in Table 2. The low values of relative standard deviation (RSD) indicate good precision and reproducibility of the methods. The average percent recoveries (Recovery%=C_foun_/C_taken_×100%) obtained were quantitative (98.80-101.33%), indicating good accuracy of the methods.

**Table 2:** Analysis of AFZ in bulk powder by BCP, BPB and BTB methods

Method	μg.ml^-1^		Relative error (%)	RSD(%)	% Range of error
	Taken	Found*			

BCP	1.20	1.19	-0.8	4.20	0.43
	5.00	4.99	-0.2	1.20	0.38
	10.0	10.0	0.0	0.80	0.40
	20.0	20.1	0.5	0.74	0.51
	35.0	35.1	0.3	0.63	0.60
BPB	0.85	0.84	-1.2	2.38	0.31
	5.00	5.02	0.4	1.39	0.46
	10.0	9.98	-0.2	0.90	0.62
	20.0	20.0	0.0	0.65	0.53
	40.0	40.1	0.2	0.47	0.44
	45.0	45.2	0.4	0.46	0.75
BTB	0.65	0.64	-1.5	3.12	0.46
	5.00	5.05	1.0	0.99	0.44
	10.0	10.1	1.0	0.79	0.39
	20.0	20.2	1.0	0.64	0.41
	30.0	30.4	1.3	0.59	0.36

* Average of six determinations.

### Application to the pharmaceutical dosage forms

The proposed methods have been successfully applied to the determination of alfuzosin hydrochloride in pharmaceutical preparations. The ingredients in the tablets did not interfere in the experiments. The results obtained with the proposed methods and shown in Table 3 were compared to the official non-aqueous titration method for alfuzosin (21) by means of *t-* and *F*-values at 95% confidence level. In all cases, the average results obtained by proposed methods and official method were statistically identical, as the difference between the average values had no significance at 95% confidence level. The proposed methods are simple, sensitive and reproducible and can be used for routine analysis of alfuzosin hydrochloride in pure form and in formulations. Proposed methods make use of simple reagents, which an ordinary analytical laboratory can afford. The commonly used additives such as starch, lactose, glucose, sodium chloride, titanium dioxide, and magnesium stearate do not interfere with the assay procedures.

**Table 3:** Determination of alfuzosin hydrochloride in dosage forms by the proposed methods and official method

Drug	Label claim (mg/tablet)	% Found^a^ + SD			
		Proposed methods			Official method (21)
		BCP	BPB	BTB	

Alfosin	2.5	100.48 ± 0.18	100.25 ± 0.19	101.02 ± 0.22	100.58 ± 0.16
		t=1.68	t=1.30	t=2.11	t=1.91
		F=1.26	F=1.41	F=1.89	
Alfosin	5	100.32 ± 0.17	100.24 ± 0.13	100.47 ± 0.15	99.68 ± 0.13
		t=1.66	t=1.12	t=1.81	t=1.35
		F=1.71	F=1.00	F=1.33	
Alfuzosin	5	99.68 ± 0.19	100.45 ± 0.16	99.70 ± 0.21	99.64 ± 0.14
		t=1.40	t=1.80	t=0.97	t=1.39
		F=1.84	F=1.31	F=2.25	

a Five independent analyses. At 95% confidence level t-value is 2.776 and F-value is 6.26.

## CONCLUSIONS

The proposed method (BCP, BPB and BTB) can be used for determination of Alfuzosin in tablets. The method is rapid, simple and has great sensitivity and accuracy. Proposed method makes use of simple reagents, which an ordinary analytical laboratory can afford. Method is sufficiently sensitive to permit determination even down to 0.18 μg ml^-1^. The proposed method is suitable for routine determination of Alfuzosin in its formulations. The commonly used additives such as starch, lactose, titanium dioxide, and magnesium stearate do not interfere with the assay procedures.
